# PP83 Inequities In Respiratory Syncytial Virus Burden Among Brazilian Infants: A Deprivation Analysis

**DOI:** 10.1017/S0266462325103000

**Published:** 2025-12-29

**Authors:** Andressa Braga, Quenia Cristina Dias Morais, Bernardo Tura, Marcelo Correia, Marisa Santos

## Abstract

**Introduction:**

Respiratory syncytial virus (RSV) is a leading cause of severe respiratory illness in infants worldwide and is associated with significant morbidity. This study evaluated inequities in RSV-related hospitalizations among Brazilian infants under one year of age. Our objective was to evaluate how socioeconomic disparities impact RSV burden and cost-effectiveness metrics for RSV vaccination strategies.

**Methods:**

A hybrid model combining a Markov framework and a decision tree was developed. The coefficient of hospitalization was estimated using the Live Birth Information System (Sistema de Informações sobre Nascidos Vivos) and the Hospital Information System (Sistema de Informações Hospitalares). RSV-related hospitalizations were identified using International Classification of Diseases, 10th Revision codes, and deprivation quintiles were defined by the BrazDep index. The RSV hospitalization risk reduction of 0.30 was extrapolated from the literature for the first year of life. Incremental cost-effectiveness ratios (ICERs) were calculated using Brazil’s cost-effectiveness threshold of USD6,873 (USD1=BRL5.82).

**Results:**

Infants in the most deprived quintile experienced 33-fold higher hospitalization rates compared with the least deprived quintile. At the current vaccine price of USD47.82, the ICER was USD26,934, exceeding the national threshold. Cost effectiveness was only achieved in the most deprived quintile, where the vaccine price threshold was USD48.29. Threshold prices for quintiles two to five were significantly lower: USD12.21, USD6.01, USD4.42, and USD2.15, respectively. Vaccination reduced hospitalization risk proportionally across quintiles, but the burden remained disproportionately high in the most deprived quintile.

**Conclusions:**

At the current price, RSV vaccination was not cost effective in most scenarios in Brazil, with ICERs exceeding the national threshold. Cost effectiveness was achieved only in the most deprived quintile, where the disease burden is highest. To align with Brazil’s threshold, significant price reductions or targeted subsidies would be necessary to ensure equitable vaccine access and public health impact.

